# Experimental Infection of *Peromyscus* Species Rodents with Sin Nombre Virus

**DOI:** 10.3201/eid2809.220509

**Published:** 2022-09

**Authors:** Kaye Quizon, Kimberly Holloway, Mahmood Iranpour, Bryce M. Warner, Yvon Deschambault, Geoff Soule, Kevin Tierney, Darwyn Kobasa, Angela Sloan, David Safronetz

**Affiliations:** Public Health Agency of Canada, Winnipeg, Manitoba, Canada (K. Quizon, K. Holloway, M. Iranpour, B.M. Warner, Y. Deschambault, G. Soule, K. Tierney, D. Kobasa, A. Sloan, D. Safronetz);; University of Manitoba, Winnipeg (D. Kobasa, D. Safronetz)

**Keywords:** Sin Nombre virus, viruses, Peromyscus, Peromyscus leucopus, Peromyscus maniculatus, rodents, Orthohantaviruses, hantavirus cardiopulmonary syndrome

## Abstract

We demonstrate that 6 distinct *Peromyscus* rodent species are permissive to experimental infection with Sin Nombre orthohantavirus (SNV). Viral RNA and SNV antibodies were detected in members of all 6 species. *P. leucopus* mice demonstrated markedly higher viral and antibody titers than *P. maniculatus* mice*,* the established primary hosts for SNV.

*Orthohantaviruses*, a genus of enveloped, segmented, negative-sense, single-stranded RNA viruses, are the cause of hantavirus cardiopulmonary syndrome. Hantavirus species are primarily associated and coevolve with specific rodent host species (*1*,*2*). In North America, Sin Nombre virus (SNV) causes most confirmed cases of hantavirus cardiopulmonary syndrome (*3*) and is primarily maintained in *Peromyscus maniculatus* deer mice (*4*). *P. maniculatus* mice are widely distributed in North America (Figure) and are a complex of subspecies that diverge according to geographic location (*2*,*7*). Likewise, SNV and its related viruses are found to diverge in association with their rodent reservoirs (*1*). Although host switching is thought to be uncommon in SNV (*1*,*2*), other rodent species sharing a geographic site were recently found to carry the virus, potentially acting as additional reservoirs and sources of human infection (*8*). We evaluated the permissiveness of 6 colony-bred *Peromyscus* mouse species, whose founders originated from locations across North America, to infection by SNV originating from New Mexico.

**Figure:**
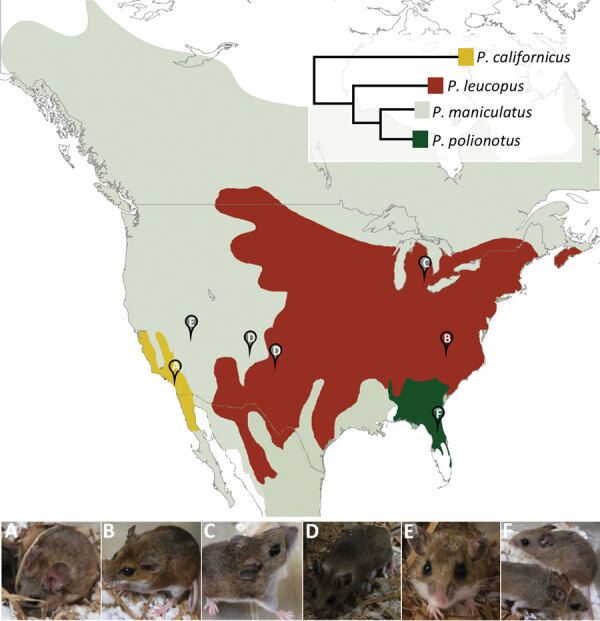
Geographic distribution of *Peromyscus* mouse species represented in a serologic analysis of serum samples from experimentally infected peromyscine rodents. Pins indicate collection sites of colony founders: *P. californicus* (A), *P. leucopus* (B)*,* *P. maniculatus bairdii* (C), *P. maniculatus rufinus* (D), *P. maniculatus sonoriensis* (E), and *P. polionotus* (F). Inset shows phylogenetic relationships with evolutionary distances estimated by Miller and Engstrom (*5*). Map adapted from Bedford and Hoekstra (*6*).

## The Study

We obtained geographically distinct peromyscine mice (of both sexes and >6 weeks of age) from breeding colonies maintained by the *Peromyscus* Genomic Stock Center, University of South Carolina (Columbia, SC, USA) (*P. maniculatus bairdii* prairie deer mouse, *P. maniculatus sonoriensis* Sonoran deer mouse, *P. polionotus subgriseus* Oldfield mouse, *P. leucopus* white-footed or wood mouse, *P. californicus insignis* California mouse), and the University of Manitoba (Winnipeg, Manitoba, Canada) (*P. maniculatus rufinus* deer mouse) (Figure) (*9*). We experimentally infected the mice with SNV strain no. 77734, which had been exclusively passaged in *P. maniculatus rufinus* mice (both the virus and the host used to propagate it originating from New Mexico) as previously described (*9*–*11*). At no point postinoculation did we note any deleterious effects of infection in rodents. We collected tissue samples (lung, heart, spleen, kidney) and either blood (days 7 and 14) or serum (days 21 and 42) samples from mice at 7, 14, 21, and 42 days postinfection (dpi) and evaluated them for the presence of viral (small-segment) RNA (*10*). We performed ELISA on serum samples, as previously described (*12*), to assess seroconversion.

We detected SNV RNA in all 6 species to varying degrees; peak median levels occurred at 21 dpi (Figure). The lowest levels of viral RNA (a surrogate metric for permissiveness and viral infection or replication) were in *P. maniculatus sonoriensis*, *P. polionotus*, and *P. californicus* mice. These results align with expectations of *P. californicus* mice, which differ genetically and geographically from *P. maniculatus* mice (Figure). However, patterns of viral detection can vary between *P. maniculatus* mice subspecies. For instance, patterns of viral RNA positivity in *P. maniculatus bairdii* mice were markedly similar to those in *P. maniculatus rufinus* mice, despite the distance between their original trapping locations. Median viral RNA peaked at 21 dpi and were sustained through 42 dpi; however, only serum was positive at 42 dpi in *P. maniculatus rufinus* mice. *P. maniculatus bairdii* mice founders were trapped in southeastern Michigan (Figure), whereas *P. maniculatus rufinus* mice descended from founders from central and northwestern New Mexico (Figure). *P. leucopus* mice appear to be highly permissive to SNV based on detection of SNV RNA. We observed high levels of viral RNA in serum, heart, kidney, lung, and spleen samples at 21 dpi and 42 dpi. Further studies are required to confirm whether this finding translates to persistent infection, viral shedding, and possible transmission between animals.

Although sex differences have been noted to have little effect on SNV infection in *P. maniculatus rufinus* mice (*10*), differences may exist in other rodent species. The numbers in our study are small; however, the data suggest sex-related differences might occur according to species. For example, male *P. maniculatus bairdii* mice have higher viral RNA titers at 42 dpi, whereas in *P. maniculatus sonoriensis* mice at 21 dpi, almost all positive animals are female. Meanwhile, *P. leucopus* mice showed indiscriminate viral RNA detection between sexes across all timepoints (Appendix Figure). Future studies are required to shed light on this aspect.

*P. maniculatus* mice were established early on as the primary reservoir for SNV (*4*). However, studies of this virus–host relationship rarely report the host taxon beyond the species level. Moreover, although levels of viral RNA tended to be negligible in the early stages of infection, an observation consistent with previous studies in susceptible animals (*9*,*10*,*13*), the detection of viral RNA at later timepoints probably means members of these species are nonetheless susceptible to SNV infection. That animals were positive at 42 dpi hints at the possibility of persistent infection, although longer-term studies would be needed to confirm.

Antibodies against the SNV nucleoprotein were detected in most serum samples collected from days 21 and 42 from the peromyscine rodents (Table). The lone exception was *P. californicus* mice, for which only a single animal had detectable SNV antibodies. Rates of seroconversion broadly mirrored the rates of detection by quantitative reverse transcription PCR. The development of antibodies reactive to the SNV nucleoprotein appeared to be delayed in male mice from 2 species (*P. maniculatus bairdii* and *P. maniculatus sonoriensis*), and only 1 of 6 of these rodents demonstrated positive serum samples at 21 dpi.

**Table:** Summary of serologic analysis of serum samples from peromyscine rodents experimentally infected with Sin Nombre virus*

Day	Sex	*Peromyscus maniculatus rufinus*	*P. m. bairdii*	*P. m. sonoriensis*	*P. leucopus*	*P. polionotus*	*P. californicus*
Day 21	M	2/3	0/3	1/3	3/4	3/3	0/3
	F	3/3	3/3	3/3	3/3	3/3	0/3
	Total	5/6	3/6	4/6	7/7	6/6	0/6
Day 42	M	3/3	3/3	3/3	3/3	4/4	1/3
	F	3/3	3/3	3/3	2/3	3/3	0/3
	Total	6/6	6/6	6/6	5/6	7/7	1/6

*Results shown are the number of seropositive animals per timepoint. Serum samples were tested by using a recombinant Sin Nombre orthohantavirus nucleocapsid-based ELISA for reactive antibodies that used a *P.* *leucopus* IgG heavy- and light-chain secondary antibody. Titers of >100 were considered positive.

## Conclusions

Overall, our data support recent observations from Goodfellow et al. (*8*) that rodents other than *P. maniculatus* mice are capable of carrying SNV without showing signs of disease. In that study, wild-caught members of *P. boylii* mice, *Mus musculus* mice, and *Tamias minimus* chipmunks trapped at 2 sites had detectable SNV RNA in their lung tissues. SNV sequences from these rodents were more similar to each other than to previously reported sequences, suggesting circulation of the virus within these rodent populations. 

In our study, we experimentally infected 6 *Peromyscus* mouse species, including 3 that fall under the *P. maniculatus* species complex, and whose founders originate from locations across North America, with a strain of SNV originating from a single wild-caught animal. Although replicating SNV was detected in animals of all 6 species, permissiveness of each species to SNV infection varied. Susceptibility to SNV infection appears to be multifactorial and is not fully explained by characteristics relating to geographic proximity or genetic relatedness. Given the variability in the patterns of infection between subspecies within *P. maniculatus*, future studies should consider reporting the subspecies when studying this virus–host relationship. Furthermore, other *Peromyscus* mouse species may play an important role in the molecular evolution and transmission of SNV. Host-switching events are thought to give rise to SNV variants and even new hantaviral species (*1*,*2*,*14*). That SNV was capable of replicating to high levels in *P. leucopus* mice for sustained periods is notable. The geographic distributions of *P. maniculatus* and *P. leucopus* mice overlap greatly, presenting opportunities for these 2 populations and their respective viruses to come into contact. Together, these regions cover most of North America, including nearly the entirety of the contiguous United States. 

These findings demonstrate the importance of broadening our understanding of the SNV–*P. maniculatus* virus–host relationship and highlight the benefit of identifying infected and infectious rodent reservoirs at the subspecies level to help elucidate epizootics and spillover events to humans. Furthermore, although distinct eastern and western patterns of genetic evolution have been documented in *P. maniculatus* rodents and associated strains of SNV in North America (*1*,*2*), our study suggests that these patterns might not necessarily prevent western lineages of SNV from emerging into eastern populations of peromyscine rodents.

Appendix Additional information about experimental infection of *Peromyscus species* rodents with Sin Nombre virus.
