# Supplementary material for: Experimental Infection of Peromyscus Species Rodents with Sin Nombre Virus
Source: Emerg Infect Dis. 2022 Sep;28(9):1882–5. doi: 10.3201/eid2809.220509 (PMC9423932; doi:10.3201/eid2809.220509)
Supplement: Appendix — Additional information about experimental infection of Peromyscus species rodents with Sin Nombre virus. [file 22-0509-Techapp-s1.pdf]

Article DOI: https://doi.org/10.3201/eid2809.222509

Experimental Infection of *Peromyscus* Species Rodents with Sin Nombre Virus

Appendix

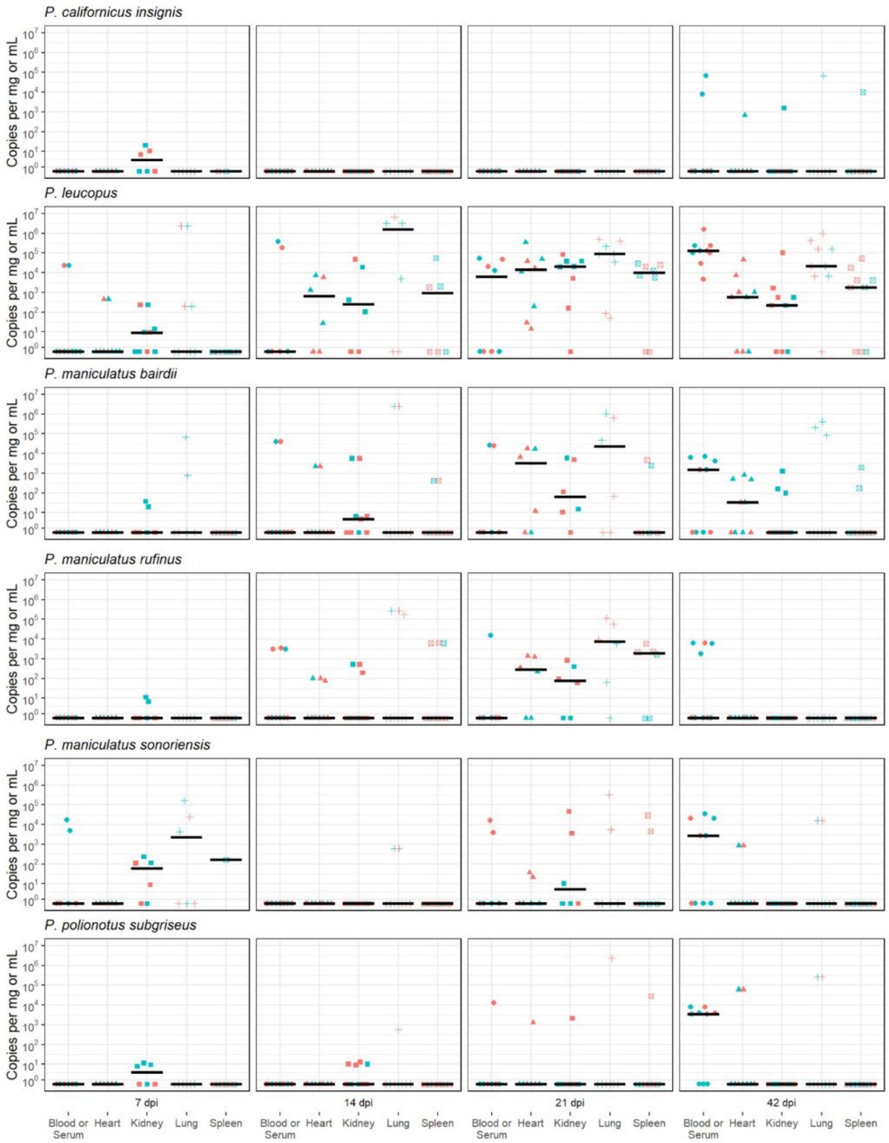

Appendix Figure. Detection of Sin Nombre orthohantavirus S segment RNA by quantitative reverse transcription PCR at 7, 14, 21, and 42 days postinfection (dpi) in various tissues. Horizontal bars denote median values. Serum samples were collected instead of blood on 21 dpi and 42 dpi for serologic analysis. Colors indicate sex: red for female, blue for male.
